# Supplementary material for: The right to water: Impact on the quality of life of rural workers in a settlement of the Landless Workers Movement, Brazil
Source: PLoS One. 2020 Jul 20;15(7):e0236281. doi: 10.1371/journal.pone.0236281 (PMC7371196; doi:10.1371/journal.pone.0236281)
Supplement: S1 File — (PDF) [file pone.0236281.s001.pdf]

Research Script **Date:**

Name: ____
Date of Birth: ___________________
Occupation: _____________________________________
School Level: __________________________________
Marital Status: _______________________________

General Questions:
How long have you lived in this camp?
Do you live alone?
How do you get income for your livelihood?
What is the main difficult you face as a rural worker?

Questions about human rights principals
Do you know that access to water and sanitation is a human right?
Who is responsible to provide access to water and sanitation in the camp?
Do you participate or have participated in any discussion about access to water and sanitation in the camp?
Do you participate or have already participated in actions of supervision and/control of the actions and decisions of the government or other actors (institutions) in relation to access to water and sanitation in the camp?
Do you know what the government and other actors (institutions) have done with regard to access to water and sanitation in the camp?

Questions about access to water:
How and where do you get water to drink, cook and take a bath?
Where do you clean your cloths and other belongings?
Where do you take a bath? How many times a week?
Who pays for the water you use?
Do you need to walk a long path to collect water?
How many times a day do you need to collect water?
Do you feel safe when go to collect water?
Are there cases of violence against women during the journey to collect water?
Do you have any idea of how much water do you use per day?
Do you think the amount of water you use daily is enough for you and your family drink, do your personal and household hygiene?
Do you use water for planting? Do you get enough water for this?
Do you think the access to water you have today impact your work?
Do you need to pay for the water you use?
Do you think that the access to water you have today affects your health in some way? How?
Do you think the access to water could improve? How?

Question about access to sanitation:
Where do you urinate and defecate?
Do you can urinate and defecate whenever you feel like or need to wait for a specific time of the day?
Do you have privacy?
Do you feel safe when need to go to urinate or defecate?
Are there cases of violence against women when they need to go out to urinate or defecate?
Do you think that the access to sanitation you have affects your health in some way? How?
Do you think the access to sanitation could improve? How?
