# Supplementary material for: The right to water: Impact on the quality of life of rural workers in a settlement of the Landless Workers Movement, Brazil
Source: PLoS One. 2020 Jul 20;15(7):e0236281. doi: 10.1371/journal.pone.0236281 (PMC7371196; doi:10.1371/journal.pone.0236281)
Supplement: S2 File — (PDF) [file pone.0236281.s002.pdf]

PARECER CONSUBSTANCIADO DO CEP

DADOS DO PROJETO DE PESQUISA

Título da Pesquisa: Direitos humanos e vulnerabilidade social: percepção da população de rua e dos trabalhadores do MST sobre o acesso à água e ao esgotamento sanitário

Pesquisador: Priscila Neves Silva

Área Temática:

Versão: 2

CAAE: 49209515.0.0000.5091

Instituição Proponente: Centro de Pesquisas René Rachou/Fundação Oswaldo Cruz/ CPqRR/

Patrocinador Principal: MINISTERIO DA EDUCACAO

DADOS DO PARECER

Número do Parecer: 1.317.762

Apresentação do Projeto:

O projeto visa obter informações sobre a percepção dos moradores de rua e trabalhadores do MST, sobre a situação deles frente ao acesso à água e esgotamento sanitário dentro da perspectivas dos Direitos Humanos.

Objetivo da Pesquisa:

O acesso à água e ao esgotamento sanitário é um direito constitucional dos cidadãos, sendo elemento fundamental para assegurar as necessidades básicas dos seres humanos. Os moradores de rua e trabalhadores do MST evidentemente são parcelas da população com carência destes benefícios sanitários.

Avaliação dos Riscos e Benefícios:

o projeto não apresenta nenhum risco para os participantes que serão submetidos a uma entrevista. Os benefícios que serão obtidos com os resultados das análises poderão apontar medidas para minimizar ou resolver as dificuldades de acesso à água e ao adequado destino dos dejetos nestas populações vulneráveis.

Comentários e Considerações sobre a Pesquisa:

É uma pesquisa original e relevante. Está muito bem elaborada do ponto de vista da fundamentação cientifica e metodologia.

Considerações sobre os Termos de apresentação obrigatória:

Os termos de apresentação obrigatória estão de acordo com as normas estabelecidas.

Recomendações:

Sem recomendações.

Conclusões ou Pendências e Lista de Inadequações:

Sem pendências ou inadequações, pois a pesquisadora apresentou o documento oficial do Movimento dos Sem Terra.

Considerações Finais a critério do CEP:

Após submissão e análise criteriosa do protocolo em questão, constamos que o estudo atende aos aspectos fundamentais da Resolução 466/2012 do Conselho Nacional de Saúde, sobre Diretrizes e Normas Regulamentadoras de Pesquisas Envolvendo Seres Humanos. Diante do exposto, o Comitê de Ética do CPqRR/FIOCRUZ Minas, de acordo com as atribuições à ele concedidas pela Legislação vigente, manifesta -se pela aprovação do projeto de pesquisa proposto. Firma-se diante deste documento a necessidade de serem apresentados os relatórios semestrais e final, bem como a notificação de eventos adversos, de emendas ou modificações no protocolo para apreciação do CEP.

Este parecer foi elaborado baseado nos documentos abaixo relacionados:

Tipo Documento	Arquivo	Postagem	Autor	Situação
Informações Básicas do Projeto	PB_INFORMAÇÕES_BÁSICAS_DO_PROJETO_569776.pdf	10/11/2015 17:06:56		Aceito
Outros	carta_aceite.pdf	02/10/2015 11:51:52	Priscila Neves Silva	Aceito
Projeto Detalhado / Brochura Investigador	ProtocolodePesquisafinal.doc	14/09/2015 16:50:31	Priscila Neves Silva	Aceito
Folha de Rosto	folha_rosto.pdf	14/09/2015 14:18:30	Priscila Neves Silva	Aceito
TCLE / Termos de Assentimento / Justificativa de Ausência	TCLE_final.docx	14/09/2015 12:37:36	Priscila Neves Silva	Aceito
Outros	carta_convite_2_MST.pdf	14/09/2015 12:33:20	Priscila Neves Silva	Aceito
Outros	carta_convite_MST.pdf	14/09/2015 12:32:41	Priscila Neves Silva	Aceito

Endereço: Avenida Augusto de Lima, 1715
Bairro: Barro Preto **CEP:** 30.190-002
UF: MG **Município:** BELO HORIZONTE
Telefone: (31)3349-7825 **Fax:** (31)3349-7825 **E-mail:** cepsh-cpqrr@cpqrr.fiocruz.br

Outros	roteiro_entrevistas.docx	14/09/2015 12:31:29	Priscila Neves Silva	Aceito
Cronograma	Cronograma_final.docx	14/09/2015 12:30:06	Priscila Neves Silva	Aceito

Situação do Parecer:
Aprovado

Necessita Apreciação da CONEP:
Não

BELO HORIZONTE, 11 de Novembro de 2015

Assinado por:
Naftale Katz
(Coordenador)
