# Supplementary material for: The right to water: Impact on the quality of life of rural workers in a settlement of the Landless Workers Movement, Brazil
Source: PLoS One. 2020 Jul 20;15(7):e0236281. doi: 10.1371/journal.pone.0236281 (PMC7371196; doi:10.1371/journal.pone.0236281)
Supplement: Attachment — Submitted filename: Response to Reviewers-1.docx [file pone.0236281.s003.docx]

Belo Horizonte, May 26, 2020

To: PloS One Journal

Dear Editor,

Prof. Ho Ting Wong

Ref. PONE-D-20-04524 – “The right to water: impact on the quality of life of rural workers in a settlement of the Landless Workers Movement, Brazil"

First of all we would like to thank you and the two reviewers of our manuscript for the contributions provided that certainly were instrumental for its improvement. They were all valued and we tried to accomodate most of them. Please, see bellow our comments to all recommendations of your letter.

**Journal Requirements:**

1. Please ensure that your manuscript meets PLOS ONE's style requirements, including those for file naming. The PLOS ONE style templates can be found at
<https://journals.plos.org/plosone/s/file?id=wjVg/PLOSOne_formatting_sample_main_body.pdf> and
<https://journals.plos.org/plosone/s/file?id=ba62/PLOSOne_formatting_sample_title_authors_affiliations.pdf>

Answer: The requirements was observed and modified as asked.

2. When reporting the results of qualitative research, we suggest consulting the COREQ guidelines: <http://intqhc.oxfordjournals.org/content/19/6/349>. In this case, please consider including more information on the number of interviewers, their training and characteristics; and if bias issues were considered. Moreover, please provide the script used in the interviews; and provide a table describing the demographic characteristics of the participants.

Answer: The information about the number of interviewers was outlined in **line 144**. The script was sent separately as supplementary material and the demographic characteristics of the participants are described in **line 144 to 146.**

3. We note that you have indicated that data from this study are available upon request. PLOS only allows data to be available upon request if there are legal or ethical restrictions on sharing data publicly. For more information on unacceptable data access restrictions, please see <http://journals.plos.org/plosone/s/data-availability#loc-unacceptable-data-access-restrictions>.

Answer: We confirm that data from this study are available upon request. This is due to ethical reasons. In the Informed Written Consent, signed by the participants, we affirm that no one will access the data collected, apart from the investigators. This is a necessary precaution to preserve the participant's anonymity, since it is possible for someone from the community to deduce from the full transcripts the identity of the participant. This was a request made by the ethic committee in order to approve the investigation. The ethic committee contact is [cepcoord.minas@fiocruz.br](mailto:cepcoord.minas@fiocruz.br) and the first author’s contact is priscila.neves31@gmail.com.

**Additional Editor Comments:**

Question: I recognize that there are typos on page 2 line 54, and page 3 line 68 (the superscript 2 and 3).

Answer: Thank you, we corrected them

Question: On the other hand, one of the reviewers suggested that the English standard of your manuscript should be improved. Please think about what can be done to improve.

Answer: We undertook a thorough review of the language of the manuscript.

**Reviewer #1:**

Question: First, the article is in need of greater theoretical engagement. Many of the quotes that the author(s) present are lacking in analysis. There should be greater theoretical analyses of the results, and these should be tied to the theoretical framework that is presented in the beginning of the manuscript.

Answer: We appreciate this comment and worked to improve the links between the theoretical framework and the analysis. For this, we have attempted to improved the section Discussion and tied it in a more precise way to the theoretical framework presented in the Introduction, mainly the HRWS.

Question: As part of this revision, please ensure that this paper is making an explicit contribution to theory; at present the contribution is unclear. One possibility would be to make a stronger theoretical connection to debates surrounding questions of sovereignty. The authors bring up food sovereignty, and I believe in this context it would be helpful to offer an analysis of how sovereignty over water, like over food or energy, are intertwined struggles within La Via Campesina, and how these various forms of sovereignty, and the threats to them, overlap, so that problems of water sovereignty overlap and condition food sovereignty, for example. The last comment is stylistic; while the article is exceptionally well-written, it would benefit from the careful attention of a native English speaker to ensure that the prose and arguments made are accessible.

Answer: As asked, we have strengthened the debate on water and food sovereignty and decided to rearrange the two paragraphs that come aftter in order to see if the idea is clear. (please see **lines 93 to 114 and 338 to 342**)

**Reviewer #2:**

Question: There is given lot of relevance for United Nations declaration, as if from this declaration on, everybody magically has access to water. I would like to suggest this tone to be a little bit changed.

Answer: We support the idea that the HRWS "can be used as a framework to regulate the access of water in several countries and have the potential of significantly contributing to public policy formulation and implementation". We introduced some changes in the text in order to made it clearer. **Lines 32 to 39**

Question: In rural areas, water is used in a much higher quantity for agriculture rather than to be used by people. Could not agree on the expression "water is also often vital for food production". Access to water in rural areas is a duty for the State in exception situations only.
Answer: We used a more nuanced language and tried to clarify our position on this issue (**line 49)**

Question: Line 83 should make reference to the 1966 Pact on Economic, Social and Cultural rights.

Answer: We included it, l**ines 67 and 68**

Question: Line 85. I did not understand if the phrase is said by the authors of the manuscript or by Metha. Anyway, I would like to know why it would be impossible to separate household uses of water from productive ones.

Answer: We tried to improve the text and to clarify this issue (**lines 69 to 77**)

Question: Line 91. Is HRWS free of charge? Would people be allowed to use (and abuse) the right to water? Neither responsibility on sustainability measures nor simply a duty to pay for the services of having treated water at home?

Answer: We clarified this point in **lines 82 to 87**

Question: The point, by and large, is that I did not see much relation between this introduction (from line 1 to 98) and the subject of the paper. Why the authors express so much about the International Right to Water (and many interpretations to it)? In line 88 it is said that the HRWS is "a framework of reference". How? And what is that for?

Answer: We expanded the explanation about the connection of the framework of the HRWS and the approach of the manuscript. At the same time, we improved the Discussion session, articulating in a clearer way with the theoretical framework.

Question: Lines 110-114 -- It would be better to say that as a hypothesis rather than as a conclusion.
Answer: We decided to delete this paragraph and rearrange the two paragraphs before. After introducing what the first reviwer asked, abour soverenignty, we thought that would be better to rearrange those paragraphs.

Question: Lines 122-155 -- Interviews with people should be authorised by an Ethic Committe, indicated at line 182. I request the authors to present that authorisation to Editorial Board of the Journal as a condition for publishing the paper.
Answer: It was submitted separately as supplementary material

Question: Lines 281 and 390-- I wonder if "malnutrition" could be substituted for "widespread hunger" or "starving"

Answer: We believe that malnutrition is a more precise wording since the population produces food, but it needs to be in a sufficient quantity and multiple variety to provide them with a minimum nutritional level.

Question: Line 313 -- "be improved" instead of "improve" sounds better.

Answer: We modified it as asked. **Line 314**

Question: Line 325 -- "as safe" should be substituted for "safe"

Answer: We modified it as asked. **Line 325**

Question: Lines 328-9 -- wording should be improved

Answer: We improved this part as asked. L**ines 328 to 331.**

Question: Lines 339-341. I haven't understood the relation authors want to do between caretaking practice and the right to water or the lack of water in the region. I ask authors to be more precise on it.

Answer: We tried to improve the arguments. **Lines 342 to 345**

Question: Line 352 -- Although it is written that "land ensures income", the paper says that it does not. Land is necessary but not sufficient (as correctly said in line 359).

Answer: We modified the text accordingly. **Lines 353 to 359**

Question: Line 374 -- I guess there is something wrong here in this expression: "community undergoing investigation"

Answer: We modified it. **Line 379**

Question: Line 397 -- Which authors have pointed that out?

Answer: We included it. **Line 405**

Question: Line 402 -- Why it should be understood this way? I would like to see authors' arguments on it.

Answer: We tried to improve the arguments. **Lines 409 to 412**

Question: Line 415 -- There as International Documents that say exactly this. See, for instance, the Vienna Declaration, adopted at the World Conference of Human Rights (2003), mainly article 5 (but feel free to quote from the first "consideranda" to articles 1-5)

Answer: We included this information. **Line 424**

Question: Line 423 -- Do States really have any legal obligation to respect, protect and fulfill human rights? There is a lot of discussion on it. Anyway, legal obligation really starts when a State internally recognises a Human Right by positing a law by which that right shall be protected. For instance, the right to health in Brasil is respected in the terms of Law 8080 (SUS), rigth to housing in the terms of Minha Casa Minha Vida Program and so on...

Answer: We tried to improve the text and to clarify this issueanswered it in **lines 432 to 435**

Question: Line 429 -- I did not understand "right to live on land, but also the right to land" From my point of view, in both cases is possible to fight for access to water, and in a very equivalent manner.

Answer: We modified it in **line 440 to 444**

Question: Lines 432-434. I didn't understand. "The use of human rights...can reduce social inequalities"? I believe that human rights can be the grounding for a public policy, but it is the public policy, duly implemented, the instrument that actually can reduce social inequalities. Human rights on their own cannot magically cause reduction of inequalities.

Answer: We modified the text in **lines 445 to 449**

Question: Line 449. It is clear that access to water is a fundamental factor to ensure life conditions, not only life with dignity!

Answer: We modified the text in **lines 463 to 464**

Question: In Conclusions, considering that authors want to demand for a public policy, they should at least say that rural exodus is a problem that is expensive and that the cost for keeping people in rural areas (furnishing water to them) is less expensive. You can only propose a public policy if Government understands that there is a lack of rights (and there is a lack of right to water) AND a social problem that is bigger and that need to be avoided. On the agenda setting of public policies, cf. John Kingdon or, in Portuguese, Celina Sousa.

Answer: We improved the arguments in **lines 474 to 477**

Hope we could answer apropriatelly all your questions and improve the paper.

Sincerelly yours,

Priscila Neves-Silva
