# Supplementary material for: The right to water: Impact on the quality of life of rural workers in a settlement of the Landless Workers Movement, Brazil
Source: PLoS One. 2020 Jul 20;15(7):e0236281. doi: 10.1371/journal.pone.0236281 (PMC7371196; doi:10.1371/journal.pone.0236281)
Supplement: Attachment — Submitted filename: Response to Reviewers_plos_260620.docx [file pone.0236281.s004.docx]

Belo Horizonte, June 26, 2020

To: PloS One Journal

Dear Editor,

Prof. Ho Ting Wong

Ref. PONE-D-20-04524 – “The right to water: impact on the quality of life of rural workers in a settlement of the Landless Workers Movement, Brazil"

Thank you and the reviewers for the contributions provided. Please, see bellow our comments to your request.

**Additional Editor Comments:**

Request: Regarding item 2 of the journal requirement, you are required to provide information related to the interviewers.
However, I find that the information in line 144 to 146 that you mentioned in your response are only related to the interviewees.

Answer: So sorry, I missed that request. I organized the information you asked, about interviewer characteristics and training, in **lines 126-131**. About consideration of bias issues, is was already described in **lines 169-175**. Script was sent with the last revision of the manuscript. Please, confirm if that is what you expected.

**Reviewers' comments:**

If I understood, about the reviewers, there is no further request.

Hope I could answered your questions properly,

Best Regards,

Priscila Neves Silva
